# Exploring the Therapeutic Potential of Three Cucurbit Plants Involving In Vivo Diabetes Screening

**DOI:** 10.7759/cureus.78861

**Published:** 2025-02-11

**Authors:** Vikas Gautam, Ashutosh Ranjan, Kumar G Bajpai, Syed Shabihe R Baqri, Anand M Saxena

**Affiliations:** 1 Department of Zoology, University of Lucknow, Lucknow, IND; 2 Department of Zoology, Shia Post Graduate College Lucknow, Lucknow, IND

**Keywords:** diabetes mellitus, luffa acutangula, momordica charantia var. muricata, traditional medicine, trichosanthes cucumerina

## Abstract

Background: *Trichosanthes cucumerina* L. (TC), *Momordica charantia* var. *muricata* (Willd.) Chakrav. (MCM), and *Luffa acutangula* (L) Roxb. (LA) are common vegetables widely used in traditional and folk systems of medicine for various ailments including diabetes.

Methodology: This work evaluated the antidiabetic potential of 95% ethanolic extract (EE) obtained from *Trichosanthes cucumerina*, *Momordica charantia *var. *muricata*,* *and *Luffa acutangula *whole plant. Their antidiabetic activity was screened in fasted, fed, glucose-loaded, and diabetic rats treated for up to 4 h.

Results: The tested herbal extracts possess potent therapeutic applications; more specifically *Momordica charantia *var.* muricata *whole plant extract possessed the most significant blood glucose lowering in fasted (19.68%; p<0.001), fed (8.1%; p<0.001), glucose loaded (18.78%; p<0.001), and diabetic (30.99%; p<0.001) models.

Conclusion: Our method of testing the glucose-lowering in four experimental models offers a time-saving approach to screen the hypoglycemic potential of candidate drugs. These findings validate the traditional claims and provide novel insights into the quest for antidiabetic cure.

## Introduction

Diabetes is considered a chronic metabolic disorder characterized by hyperglycemia (increasing fasting and postprandial blood glucose) and carbohydrate, fat, and protein metabolism imbalances leading to several complications due to insulin resistance [1]. Since ancient times, herbal medicines (HMs) have played a key role in the global healthcare system. To ensure efficacy and safety, it is crucial to conduct a thorough examination of the quality and control of its diverse chemical makeup. Phytonutrients or phytochemicals, usually present in vegetables, have remained a less explored arena in the field of health management. They help plants combat disease-causing bacteria, fungi, insects, and other environmental stressors [2]. Further, they are also efficient protein modulators, intracellular signal cascade system activators, and intercalating agents because of the distinct chemical variations present in their structure [3]. For many years, natural products have been the primary source of biologically active compounds used for drug formulation and health improvement. Traditional and folk medicine knowledge has been more thoroughly studied via an ethnopharmacological approach to provide helpful insights for drug discovery and development. This has led to the discovery of several plant-derived phytomedicine. These include paclitaxel, vinblastine, vincristine, morphine, reserpine, and digoxin [4]. The incidence of obesity, cardiovascular disease, cancer, diabetes, and other chronic diseases has increased as an outcome of consuming inadequate diets, which had a large epidemiological impact on the morbidity and mortality profile of populations [5]. Microconstituents, known as antioxidants, are involved in both the scavenging of free radicals and the inhibition of lipid peroxidation, thus preventing the onset or progression of oxidative chain reactions [6].

Vegetables such as gourds are eaten all around the world and are additionally utilized widely in the Indian Ayurveda system [7]. *Luffa acutangula* (ridge gourd), *Momordica charantia* var. *muricata* (wild bitter gourd), and *Trichosanthes cucumerina* (snake gourd) are annual climbers commonly found in India, China, and other Asian countries, popularly cultivated for its fruits and have broad range of pharmacological activities such as antibacterial, antioxidants, anticancerous, antidiabetic, and antiulcerous [8-10]. The toxicity studies of tested plants proved no toxicity and deaths examined at various doses specifically at 2000 mg/kg level *Luffa acutangula* has no mortality or toxicity [11] similarly *Trichosanthes cucumerina* whole plant extract showed no mortality in chronic toxicity studies (unpublished data) and our analysis on *Momordica charantia* var. *muricata* whole plant extract acute toxicity studies showed no mortality or significant differences in organ or body weight of albino rats [12].

Plants have traditionally been used as medicines by various ethnic groups and tribes across the globe to cure a diverse set of diseases [13]. For instance, fruits, leaves, and roots of *Trichosanthes cucumerina* are used by Santhal communities of Handipua village to treat diabetes [14]. Similarly, tribes in Kerala cook fruits of *Momordica charantia* var. *muricata* as vegetables and its consumption has been shown to have sugar lowering effect [15].* Luffa acutangula* which as an appetizer also provides a low-calorie diet and is considered good for diabetes [16]. Its fruit is widely used by the local inhabitants of the reserve forest of Mahadevpur, Telangana, for the treatment of diabetes [17].

Due to the health-enhancing potential attributed to Cucurbitaceae species, this study was conducted. The findings may serve as a foundation for identifying novel and potentially bioactive compounds in these underexplored plant species [18]. To date, no studies have been conducted on the antidiabetic activities of *Momordica charantia* var. *muricata*. Furthermore, to our knowledge, no prior research has been published on the comparative potential of *Trichosanthes cucumerina*, *Momordica charantia* var. *muricata*, and *Luffa acutangula* plants. In the present study, we explored the potential of three Cucurbitaceae plant species (*Luffa acutangula* Roxb., *Trichosanthes cucumerina* L., and *Momordica charantia* var. *muricata*) for antidiabetic screening.

## Materials and methods

Chemicals and reagents

Streptozotocin (CAS no. 18883-66-4) and ethanol (CAS no. 64-17-5) were procured from Sigma-Aldrich (St. Louis, MO), and Gum Acacia Powder (CAS no. 9000-01-5) was purchased from HiMedia Laboratories Private Limited (Mumbai, India). Sodium citrate, citric acid, and sucrose were purchased from Sisco Research Laboratories Private Limited (Mumbai, India). An Accu-Chek Active glucometer manufactured by Roche Diagnostic (Rotkreuz, Switzerland) was used to monitor the blood sugar levels. All the solvents used for this work were of standard analytical grade.

Collection of plant samples

Whole plants of the family Cucurbitaceae namely *Luffa acutangula* Roxb., *Momordica charantia* var. *muricata*, and *Trichosanthes cucumerina* L., were collected in June-July 2022 from Daliganj (coordinate: 26°52’N, 80°55’E), Malihabad (coordinate: 26°54’N, 80°42’E), and Aishbagh (coordinate: 26°50’N, 80°54’E) areas of the Lucknow, India. The species were first verified by World Flora Online (ID: wfo-0000358861, ID: wfo-0000376899, ID: wfo-0000408012), also validated by the database http://www.theplantlist.org, and then authenticated by the botanist expert, Prof. Alka Kumari, Department of Botany, University of Lucknow, Uttar Pradesh, India. The collected plants were washed thoroughly and kept in shade at room temperature for drying and preparation of crude extract. The completely dried plants were then stored separately in airtight containers for further studies.

Preparation of extract

The dried plant materials were mechanically powdered and stored in containers with proper labeling (Botanical name, common name, family, date of collection, collected by, and supervisor) details. Each of these plant samples was macerated and extracted by soxhlet apparatus with 95% ethanol according to the method described by Anandika et al. [19]. Under carefully regulated conditions, the extract was concentrated using a rotatory evaporator at 40°C and dried out by evaporation in a water bath. For further research, this dried extract was stored in an airtight container.

Procurement and maintenance of animals

**Figure 1:**
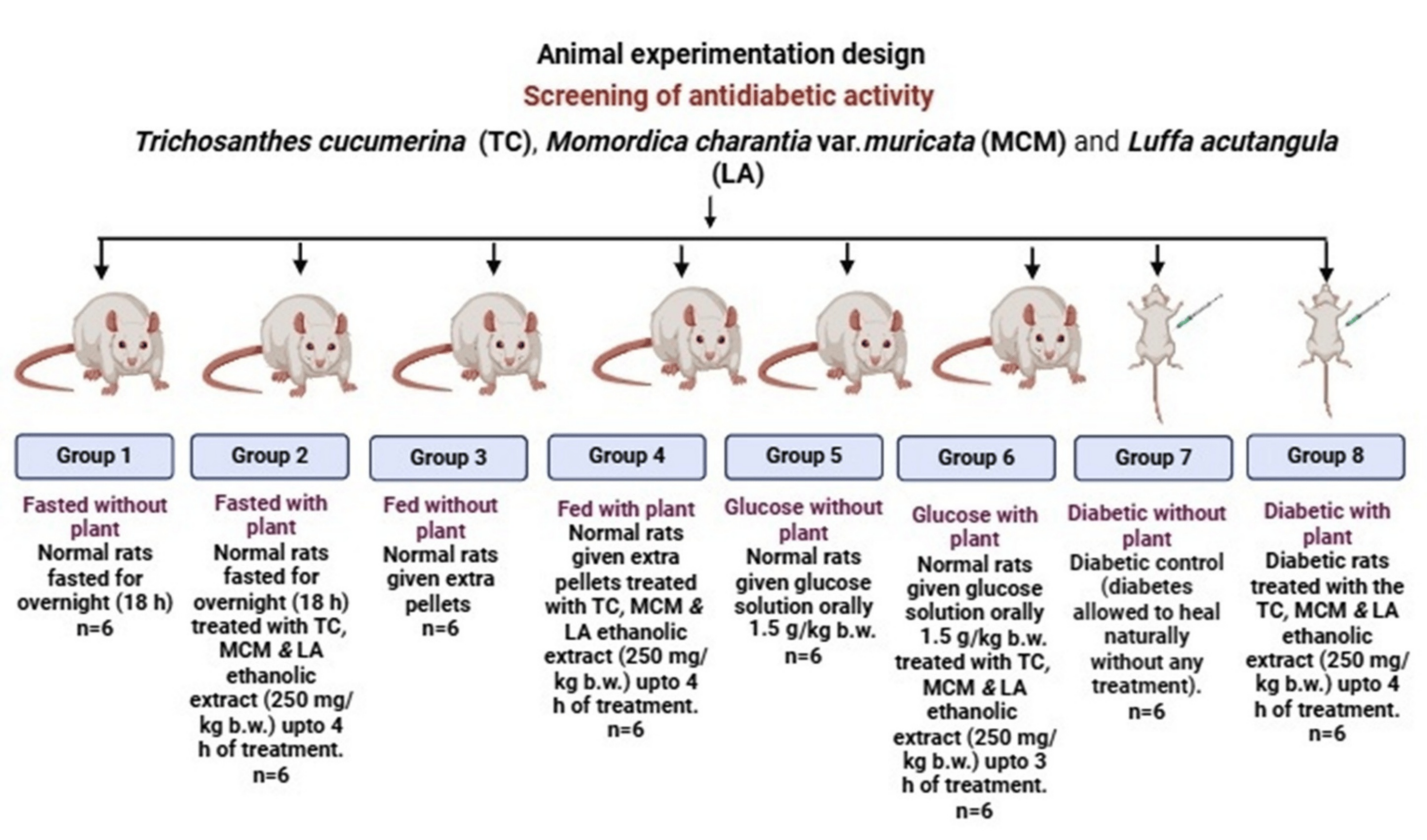
Experimental design for screening the antidiabetic activity of selected plants.

In the present study, healthy male albino rats (*Rattus norvegicus*) of Sprague Dawley (SD) strain (120-150 g) were used and procured from the National Laboratory Animal Facility, Central Drug Research Institute (CDRI), Lucknow, India. Guidelines for Animal Husbandry and Animal Ethics were strictly followed and carried out according to the Animal Research: Reporting of In Vivo Experiments (ARRIVE) [20] and Committee for the Purpose of Control and Supervision of Experiments on Animals (CPCSEA) guidelines [21]. The animals were kept in the following controlled conditions: temperature 25-26°C, relative humidity 60-70%, and 12/12 h light/dark cycle. The rats were divided into experimental (with plant extract) and control (without plant extract) groups of six rats each (Figure 1). Water was provided ad libitum. One week prior to the study, the animals were acclimatized to the laboratory environment as per Organization for Economic Co-operation and Development (OECD) guidelines [22]. The animals were divided into eight groups, randomly.

Experimental protocol

**Figure 2:**
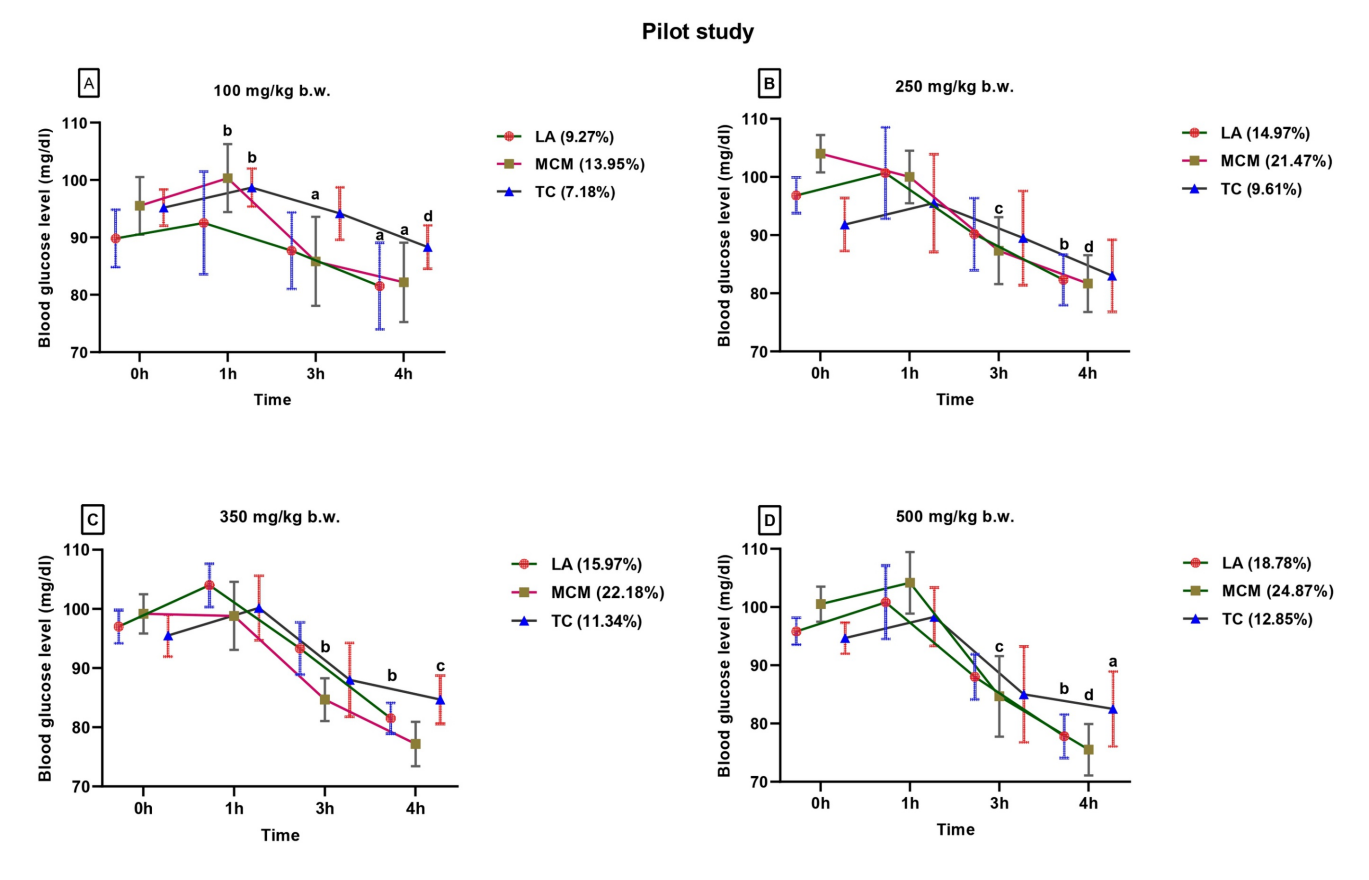
Diabetes in vivo pilot screening of Luffa acutangula (LA), Momordica charantia var. muricata (MCM), and Trichosanthes cucumerina (TC) at different doses (100, 250, 350, and 500 mg/kg b.w.). Significance for 0 h, 1 h, 3 h, and 4 h are as follows: ^a^p<0.05, ^b^p<0.01, ^c^p<0.001, and ^d^p<0.0001.

To determine the effective dose of *Luffa acutangula*, *Momordica charantia* var. *muricata*, and *Trichosanthes cucumerina*, a pilot study on different doses (100, 250, 350, and 500 mg/kg body weight) was conducted (Figures 2A-2D). On the basis of the results obtained along with toxicity considerations, the dose of 250 mg/kg body weight was selected for all the experiments conducted in the present study. The blood sugar-lowering effect of the crude extract with a single dose of 250 mg/kg body weight was examined in the four different experimental models mentioned below according to the method described by Anandika et al., while the control group was given only 2% gum acacia (vehicle) suspension to increase the sensitivity of the experimental procedure [19].

Fasted Model

Blood was collected initially (at 0 h) from the tail vein of rats fasted overnight (18 h) to estimate the glucose concentration, after which a single dose of 250 mg/kg body weight of the plant extract was administered. Once again, blood samples were collected at 1-, 3-, and 4-h intervals after feeding the extract. The blood glucose concentrations of all these samples were estimated by using the standard glucometer (Roche, Germany: Accu-Chek Active glucometer).

Fed Model

Excess pellets (Maharashtra, India: VRK Nutritional Solutions animals feed) were kept in the cages the previous evening, ensuring that some pellets were left the next morning. Blood was collected (at 0 h) and after the administration of the extract at a dose of 250 mg/kg body weight at 1-, 3- and 4-h intervals for glucose estimation.

Glucose-Loaded Model

Animals were fasted overnight (18 h) and blood was collected (at 0 h) for glucose estimation. The plant extract was then administered, and thirty minutes later, a glucose solution (1.5 g/kg body weight, oral) was given using an oral cannula (18G×50 mm). Blood samples were collected at half-hour, 1-h, and 3-h intervals for glucose estimation.

Diabetic Model

Diabetes was induced in rats by streptozotocin (35 mg/kg b.w.) dissolved in the freshly prepared chilled citrate-phosphate buffer (pH 4.5) through intra-peritoneal (i.p.) injection. Before giving injection the rats were fasted for 18 h. All rats were returned to their cages with free access to food and water after the injection of streptozotocin (STZ). To avoid transient hypoglycemia, all the rats were given 10% sucrose solution for 24 h. Three days after intra-peritoneal injection of streptozotocin, the blood glucose concentration of the rats was measured. Rats with a fasting blood glucose level over 200 mg/dL were considered diabetic and were used in this group for the experiment [23]. The same protocol we have done in the fasted group was followed and the blood glucose concentration of the rats in this group was measured at different intervals.

In all four test models, separate control experiments were carried out simultaneously using the identical protocol, except that only the vehicle (i.e., 2% gum acacia suspension) was fed to the rats.

Statistical analysis

All the data were statistically evaluated and the results are expressed as the mean±standard deviation (SD) (n=6). Comparisons among the groups were made by one-way ANOVA, followed by a post-Tukey’s multiple comparison test, and GraphPad Prism version 8.0 (San Diego, CA: GraphPad Software, LLC) was used for statistical analysis. Significance between control and experimental groups was considered significant at p<0.05, p<0.01, and p<0.001.

## Results

In the *Trichosanthes cucumerina* plant antidiabetic screening, the blood glucose levels of the control groups were measured, i.e., without plant extract treatment, there was no significant decrease, with a maximum of 1.43% at the 4th h from the initial value in the fasted model (Table 1). However, in the experimental groups treated with the *Trichosanthes cucumerina* plant extract, a significant decrease in blood glucose was detected in fasted (9.12%, p<0.001), fed (8.59%, p<0.01), glucose-loaded (4.21%, p<0.001) and diabetic (13.21%, p<0.001) models, possibly because of its ability to improve insulin sensitivity or modulate glucose absorption and metabolism (Table 2). In the case of *Momordica charantia* var. *muricata*, the blood glucose level of fasted rats without plant extract decreased by 4.4% at 4th h of treatment from the initial value, whereas the experimental group treated with plant extract presented a significant decrease in blood glucose at the 4th h which was 19.68%. The consistency in lowering blood glucose levels was observed across other models of *Momordica charantia* var. *muricata *screening, including fed (18.28%, p<0.001), glucose-loaded (18.78%, p<0.001), and diabetic (30.99%, p<0.001) models, compared with their respective controls. This suggests that *Momordica charantia *var. *muricata *may enhance glucose tolerance and insulin sensitivity, potentially benefiting the management of both fasting and postprandial hyperglycemia. It shows promise as an antidiabetic agent, particularly for managing elevated blood glucose levels under diabetic conditions. Similarly, the *Luffa acutangula* screening revealed a significant decrease in blood glucose in the plants in the experimental groups compared with the corresponding controls. The extract showed promising results in the fasted (9.69%, p<0.01), fed (4.77%, p<0.001), glucose (6.29%, p<0.001), and diabetic (21.2%, p<0.001) models indicating its ability to reduce blood glucose levels under different conditions and highlighting its potential as a natural agent for glucose diabetes management. Based on these findings, we recommend that people with diabetes should consider these plant-based foods in their daily diet, and lifestyle modifications such as regular physical activity along with maintaining a balanced diet will help improve insulin sensitivity and reverse the onset of diabetes.

**Table 1:** Effect of Trichosanthes cucumerina, Momordica charantia var. muricata, and Luffa acutangula plant on blood glucose levels in fasted, fed, and streptozotocin-induced diabetic male albino rats. Statistical significance: *p<0.05, **p<0.01, ***p<0.001.

Groups	Treatment	Blood glucose level mg/dL (mean±SD) at time (hours)				Maximum % of blood glucose lowering from initial value
		0 h	1 h	3 h	4 h	
Fasted model	Control	92.83±4.22	93.83±2.79	95.33±1.37	91.5±2.07	1.43% at 4th h
	Trichosanthes cucumerina	87.67±3.27	91.7±3.06	82.83±1.72^**^	79.67±2.88^***^	9.12% at 4th h
	Control	104±5.76	112.67±4.46	108.17±8.47	99.33±5.47	4.4% at 4th h
	*Momordica charantia* var. *muricata*	106.67±7.74	100.5±9.52	86.67±7.76^***^	85.67±4.63^***^	19.68% at 4th h
	Control	86.5±5.68	87.33±2.73	88.83±4.26	87.17±3.08	3.66% 4th h
	Luffa acutangula	98.0±3.79	102.67±6.47	94.83±5.34	88.50±4.97^***^	9.69% 4th h
Fed model	Control	136.5±9.27	144.33±6.98	140.5±8.62	141.17±11.58	No lowering
	Trichosanthes​​​​​​​ cucumerina	139.67±6.25	155.0±15.49	138.67±8.24	127.67±13.5^**^	8.59% 4th h
	Control	121.67±2.42	129.67±6.22	126.33±5.01	121.17±7.14	0.4% at 4th h
	*Momordica charantia* var. *muricata*	118.5±2.43	124.67±7.09	100.67±11.06^**^	96.83±5.04^***^	18.28% at 4th h
	Control	147.7±6.53	151.83±3.49	146.17±7.49	144.83±4.88	1.94% at 4th h
	Luffa acutangula	143.0±5.72	153.33±6.47	136.17±5.04^***^	137.0±6.96^***^	4.77% at 3rd h
Diabetic model	Control	208.17±4.71	206.33±4.2	209.0±3.33	207.83±4.17	0.88% 1th h
	Trichosanthes​​​​​​​ cucumerina	206.83±2.93	196.0±4.38^*^	178.0±6.9	179.5±7.34^***^	13.21% 4th h
	Control	209.5±4.84	210.33±6.09	205.17±5.74	206.67±5.96	1.67% at 3rd h
	*Momordica charantia* var. *muricata*	208.67±5.61	196.67±8.59^***^	154.17±7.63^***^	144.67±11.64^***^	30.99% at 4th h
	Control	209.67±3.14	209.83±3.92	207.5±3.39	211.33±5.65	No lowering
	Luffa acutangula	207.5±4.18	191.5±3.45	174.0±8.25	163.33±7.2^***^	21.2% at 4th h

**Table 2:** Effect of Trichosanthes cucumerina, Momordica charantia var. muricata, and Luffa acutangula plant on blood glucose level of glucose loaded male albino rats. Statistical significance: *p<0.05, **p<0.01, ***p<0.001.

Groups	Treatment	Blood glucose level mg/dL (mean±SD) at time (hours)				Maximum % of blood glucose lowering from initial value
		0 h	Half hour	1 h	3 h	
Glucose loaded model	Control	101.5±4.76	112.33±5.57	107.17±6.11	100.67±7.81	0.81% 4th h
	Trichosanthes cucumerina	99.0±5.1	108.0±4.15	100.17±3.43	94.83±4.54^**^	4.21% 4th h
	Control	112.5±5.09	124.33±5.79	126.83±6.43	118.83±5.04	No lowering
	*Momordica charantia* var. *muricata*	95.83±10.53	116.33±14.02	89±14.56	77.83±13.47^***^	18.78% at 4th h
	Control	91.33±6.77	106.5±4.93	102.17±4.12	98.67±3.39	No lowering
	Luffa acutangula	95.33±4.37	104.67±4.59	96.5±3.78^*^	89.33±3.67^***^	6.29% at 4th h

## Discussion

The comparative analysis of the *Trichosanthes cucumerina*, *Momordica charantia* var. *muricata*, and *Luffa acutangula* ethanolic extracts demonstrate a range of blood glucose-lowering effects on different models (fasted, fed, glucose, and diabetic), where *Momordica charantia* var. *muricata* consistently resulted in the highest lowering of blood glucose in all the models, justifying its traditional role in treating diabetes and its associated complications (Table 1). In the fasted state, both *Trichosanthes cucumerina* and *Luffa acutangula* had comparable effects (9.12% and 9.69% lower glucose, respectively), however; these effects were lower than those in the fasted *Momordica charantia* var. *muricata*-treated rats (19.68%). Under fed conditions, these extracts significantly lowered the blood glucose levels, whereas *Momordica charantia* var. *muricata* again demonstrated the highest reduction (18.28%) compared with *Trichosanthes cucumerina* (8.59%) and *Luffa acutangula* (4.77%). For the glucose-loaded condition, the decrease in blood glucose indicates the postprandial efficacy, with *Momordica charantia* var. *muricata* (18.78%) again marked the highest lowering when compared with *Trichosanthes cucumerina* (4.21%) and *Luffa acutangula* (6.29%) (Table 2). Finally, *Momordica charantia* var. *muricata* caused the highest and most significant blood glucose lowering in the streptozotocin-induced diabetic (30.99%) model compared with *Trichosanthes cucumerina* (13.21%) and *Luffa acutangula* (21.2%). Of all the four experimental groups, the glucose-lowering effect of *Momordica charantia *var. *muricata* on diabetic rats is most relevant for establishing the therapeutic potential of these plants. Crude extracts contain a complex mixture of phytochemicals, including alkaloids, flavonoids, terpenoids, and phenolics, among others. Compared with isolated compounds, these compounds often work synergistically, potentially enhancing therapeutic potential [24].

The consumption of phytochemicals, particularly polyphenol compounds, and their main dietary sources may help reduce insulin resistance and associated diabetes risk factors including inflammation and oxidative stress [25]. Bahadoran et al. conducted a three-year longitudinal research on cholesterol and blood glucose levels in Tehran with 1141 people [26]. They investigated the connection between insulin resistance and the dietary phytochemical index (DPI) and reported that those with greater dietary phytochemical index were far less likely to be insulin resistant and to have hyperinsulinemia [26]. These outcomes were associated with increased intake of foods rich in phytochemicals. Diets containing vegetable food are high in phytochemicals and have the benefit of preventing obesity, which is one of the major risk factors for the development of multiple chronic diseases, including diabetes, cardiovascular disease, cancer, and chronic respiratory disorders. In a meta-analysis involving 12 clinical trials with 1151 subjects, Huang et al. examined the effects of vegetarian diets that promoted the highest weight loss when compared with a non-vegetarian diet (lost an average of 2.02 kg), suggesting that vegetarian diets significantly reduce weight [27]. A study by Juma et al. shows the dose-dependent glucose-lowering of *Luffa acutangula* methanolic fruit extract observed in Swiss albino mice [28]. While the study shows promising results, several key limitations need to be addressed to have a broader view of diabetes drug screening. In the present study, the blood glucose measurement at different time intervals (0, 1, 3, and 4 h) limits our understanding of how these plant extracts affect glucose regulation over the long term. Moreover, the animal models provide valuable preliminary insights, but further validation in human clinical trials is needed to confirm its efficacy for diabetes management.

## Conclusions

Despite numerous breakthroughs in our understanding of mechanisms that lead to faulty sugar metabolism, diabetes continues to be a global health challenge. None of the existing drugs and treatment strategies can guarantee a cure without unnecessary complications. Given a relatively safer profile of herbal medicines, exploration of compounds with hypoglycemic activity in the vast array of phytochemicals has been triggering intense research leading to very exciting results. The present research is a step in this direction which is likely to open up new possibilities for the prognosis of diabetes. Contrary to the existing screening strategies for new candidate drugs which span over several weeks, the novel approach of using four experimental models simultaneously requires a much shorter time course, thereby saving a lot of time. Besides, the use of in vivo testing of prospective drugs for metabolic disorders offers a definite advantage over in vitro studies because of the generalized effects of altered glucose metabolism, which affects most of the cells in the body. Diabetes affects the overall quality of life because of its impact on oxidative metabolism which is implicated in processes like wound healing and aging.

Herbs have traditionally been used as potent antidiabetic medications notwithstanding that the mechanisms responsible for their medicinal properties remained largely unknown. The results of this study suggest a promising therapeutic potential for *Momordica charantia* var. *muricata* which is remarkable in several ways. Deriving medicinal benefits from a common vegetable like *Momordica charantia* var. *muricata* drastically reduces the heavy cost of treatment and are major factor in diabetes management, especially in poor countries. Besides releasing the socioeconomic burden, such a treatment is also expected to save the patient from unwanted complications of synthetic drugs and even insulin. However, there is a need to further explore the therapeutic potential of *Momordica charantia* var. *muricata* and similar potential drugs so as to identify their active principles and mechanisms of their medicinal effects. In the face of the rapid advances taking place in molecular biology and genomics, future studies should be able to identify better cures for diabetes based on their interaction with molecular targets of the diseases.

## References

[1] Ghadge AA, Kuvalekar AA (2017). Controversy of oral hypoglycemic agents in type 2 diabetes mellitus: novel move towards combination therapies. Diabetes Metab Syndr.

[2] Patil SV, Borase HP, Patil CD, Salunke BK (2012). Biosynthesis of silver nanoparticles using latex from few euphorbian plants and their antimicrobial potential. Appl Biochem Biotechnol.

[3] Arts IC, Hollman PC (2005). Polyphenols and disease risk in epidemiologic studies. Am J Clin Nutr.

[4] Cragg GM, Newman DJ (2013). Natural products: a continuing source of novel drug leads. Biochim Biophys Acta.

[5] Ruel J, Ruane D, Mehandru S, Gower-Rousseau C, Colombel JF (2014). IBD across the age spectrum: is it the same disease?. Nat Rev Gastroenterol Hepatol.

[6] Irshad M, Zafaryab M, Singh M, Rizvi MM (2012). Comparative analysis of the antioxidant activity of Cassia fistula extracts. Int J Med Chem.

[7] Saha SS, Ghosh M (2011). Antioxidant effect of vegetable oils containing conjugated linolenic acid isomers against induced tissue lipid peroxidation and inflammation in rat model. Chem Biol Interact.

[8] Shendge PN, Belemkar S (2018). Therapeutic potential of Luffa acutangula: a review on its traditional uses, phytochemistry, pharmacology and toxicological aspects. Front Pharmacol.

[9] Kirana H, Srinivasan BP (2008). Trichosanthes cucumerina Linn. improves glucose tolerance and tissue glycogen in non insulin dependent diabetes mellitus induced rats. Indian J Pharmacol.

[10] Sagor AT, Chowdhury MR, Tabassum N, Hossain H, Rahman MM, Alam MA (2015). Supplementation of fresh ucche (Momordica charantia L. var. muricata Willd) prevented oxidative stress, fibrosis and hepatic damage in CCl4 treated rats. BMC Complement Altern Med.

[11] Arunachalam A, Selvakumar S, Jeganath S (2012). Toxicological studies on ethanol extract of Luffa acutangula in albino Wistar rats. Int J Curr Pharm Clin Res.

[12] Kongtun S, Jiratchariyakul W, Kummalue T, Tan-ariya P, Kunnachak S, Frahm AW (2009). Cytotoxic properties of root extract and fruit juice of Trichosanthes cucumerina. Planta Med.

[13] Rahmatullah M, Biswas A, Haq WM, Seraj S, Jahan R (2012). An ethnomedicinal survey of cucurbitaceae family plants used in the folk medicinal practices of Bangladesh. Chron Young Sci.

[14] Tripathy PK, Kumar S, Jena PK (2014). Assessment of food, ethnobotanical and antibacterial activity of Trichosanthes cucumirina L.. Int J Pharm Sci Res.

[15] Joseph JK, Antony VT (2008). Ethnobotanical investigations in the genus Momordica L. in the Southern Western Ghats of India. Genet Resour Crop Evol.

[16] Anitha J, Miruthula S (2014). Traditional medicinal uses, phytochemical profile and pharmacological activities of Luffa acutangula Linn.. Int J Pharmacogn.

[17] Rajesham K, Rao N, Venkateshwarlu M, Sammaiah D, Anitha U, Ugandhar T (2013). Studies on the medicinal plant biodiversity in forest ecosystem of Mahadevpur forest of Karimnagar (A.P.) India. India Biosci Disc.

[18] Mukherjee PK, Singha S, Kar A (2022). Therapeutic importance of Cucurbitaceae: a medicinally important family. J Ethnopharmacol.

[19] Anandika S, Vikas G, Saxena AM, Dharamveer P, Sanjay K (2022). Evaluation of the anti-diabetic potential of Rumex vesicarius L. in normal and streptozotocin induced diabetic rats. Res J Biotech.

[20] Sert NP, Ahluwalia A, Alam S (2020). Reporting animal research: explanation and elaboration for the ARRIVE guidelines 2.0. PLoS Biol.

[21] Rehman HU, Ullah K, Rasool A (2023). Comparative impact of streptozotocin on altering normal glucose homeostasis in diabetic rats compared to normoglycemic rats. Sci Rep.

[22] Qadri SS, Ramachandra SG (2018). Laws, regulations, and guidelines governing research animal care and use in India. Laboratory Animals (Second Edition).

[23] (2011). Guide for the Care and Use of Laboratory Animals. Eighth Edition.

[24] Sarker SD, Nahar L, Kumarasamy Y (2007). Microtitre plate-based antibacterial assay incorporating resazurin as an indicator of cell growth, and its application in the in vitro antibacterial screening of phytochemicals. Methods.

[25] Guasch-Ferré M, Merino J, Sun Q, Fitó M, Salas-Salvadó J (2017). Dietary polyphenols, mediterranean diet, prediabetes, and type 2 diabetes: a narrative review of the evidence. Oxid Med Cell Longev.

[26] Bahadoran Z, Mirmiran P, Tohidi M, Azizi F (2015). Dietary phytochemical index and the risk of insulin resistance and β-cell dysfunction: a prospective approach in Tehran lipid and glucose study. Int J Food Sci Nutr.

[27] Huang RY, Huang CC, Hu FB, Chavarro JE (2016). Vegetarian diets and weight reduction: a meta-analysis of randomized controlled trials. J Gen Intern Med.

[28] Juma A, Pervin R, Al Azad S (2013). Antihyperglycemic and antinociceptive activity of methanolic extract of Luffa acutangula fruits. Adv In Nat Appl Sci.

